# Audit of Nasal Bone Fracture Referrals to an ENT Hub: Timeliness of Review and Management

**DOI:** 10.7759/cureus.92814

**Published:** 2025-09-20

**Authors:** Jonathan McAdam

**Affiliations:** 1 General Surgery, Belfast Health and Social Care Trust, Belfast, GBR

**Keywords:** manipulation of nasal bones, nasal bone fracture, rapid access clinic, telemedicine services, telephone triage

## Abstract

Introduction: Nasal bone fractures (NBFs) are among the most common facial injuries and often require prompt evaluation and intervention to optimise functional and cosmetic outcomes. This audit evaluates the timeliness of review and management for patients referred to an ENT hub via email.

Methods: A retrospective review was conducted of all referrals to the ENT hub email at Ulster Hospital, Dundonald, between January 1, 2022, and March 18, 2022. All suspected NBF referrals were included. Data on demographics, time intervals from injury to referral, referral to review, and time to manipulation of nasal bones (MNB) were collected.

Results: Fifty-six referrals were identified (age range: 7-89 years). All patients were contacted via telephone triage, which reduced clinic attendances by 39%. Ninety-three percent of referrals were received within seven days of injury; however, only 63% were contacted within seven days of referral. Of the 34 patients reviewed in clinic, 82% were seen within two weeks of injury. Nineteen patients underwent MNB, with 68% receiving intervention within 14 days. All three paediatric patients requiring MNB were managed outside the 14-day window due to theatre delays.

Conclusion: Telephone triage effectively reduced unnecessary clinic reviews, but delays in contacting patients, particularly paediatric cases, affected timely management. Earlier in-clinic review for children, better communication strategies, and ensuring accurate referral information may improve service delivery.

## Introduction

Nasal bone fractures (NBFs) are the most common type of facial fracture, accounting for approximately one-third of all facial skeletal injuries [1]. They frequently occur as a result of interpersonal violence, sporting activities, falls, or road traffic collisions [2]. Although many cases are minor and may not require surgical intervention, timely assessment is crucial, as manipulation under anaesthesia (MNB) is ideally performed within 7-14 days of injury to optimise outcomes [3].

Untreated or delayed treatment of NBFs can result in cosmetic deformity, nasal obstruction, or septal deviation. Paediatric cases are particularly challenging due to their likely requirement for general anaesthesia, ongoing nasal growth, and increased risk of long-term sequelae [4].

In the United Kingdom, ENT services are increasingly centralised, with many hospitals adopting referral hubs to streamline patient pathways. At Ulster Hospital, Dundonald, referrals for suspected NBFs are submitted electronically to an ENT hub. Patients are triaged via telephone consultation, with only selected cases invited for face-to-face review. This system aims to reduce unnecessary clinic visits, but there is concern that delays in contact or clinic scheduling may impact time-sensitive management.

This audit aimed to evaluate the timeliness of referral processing, patient contact, clinic review, and intervention for suspected NBFs, and to compare performance with accepted standards.

## Materials and methods

This was a retrospective audit conducted at Ulster Hospital, Dundonald, between January 1, 2022, and March 18, 2022. The audit standard was based on ENT UK and BAOMS guidance, which recommends that patients requiring reduction of NBFs should ideally be assessed and managed within 7-14 days of injury [3].

Inclusion criteria

All referrals sent to the ENT hub email with suspected NBF during the audit period were included. No referrals were excluded. Data sources included referral emails, clinic records, and operative notes.

Data collection

Demographics, mechanism of injury, time from injury to referral, time from referral to patient contact, time from injury to clinic review, and whether MNB was required, including time from injury to MNB, were collected.

**Figure 1:**
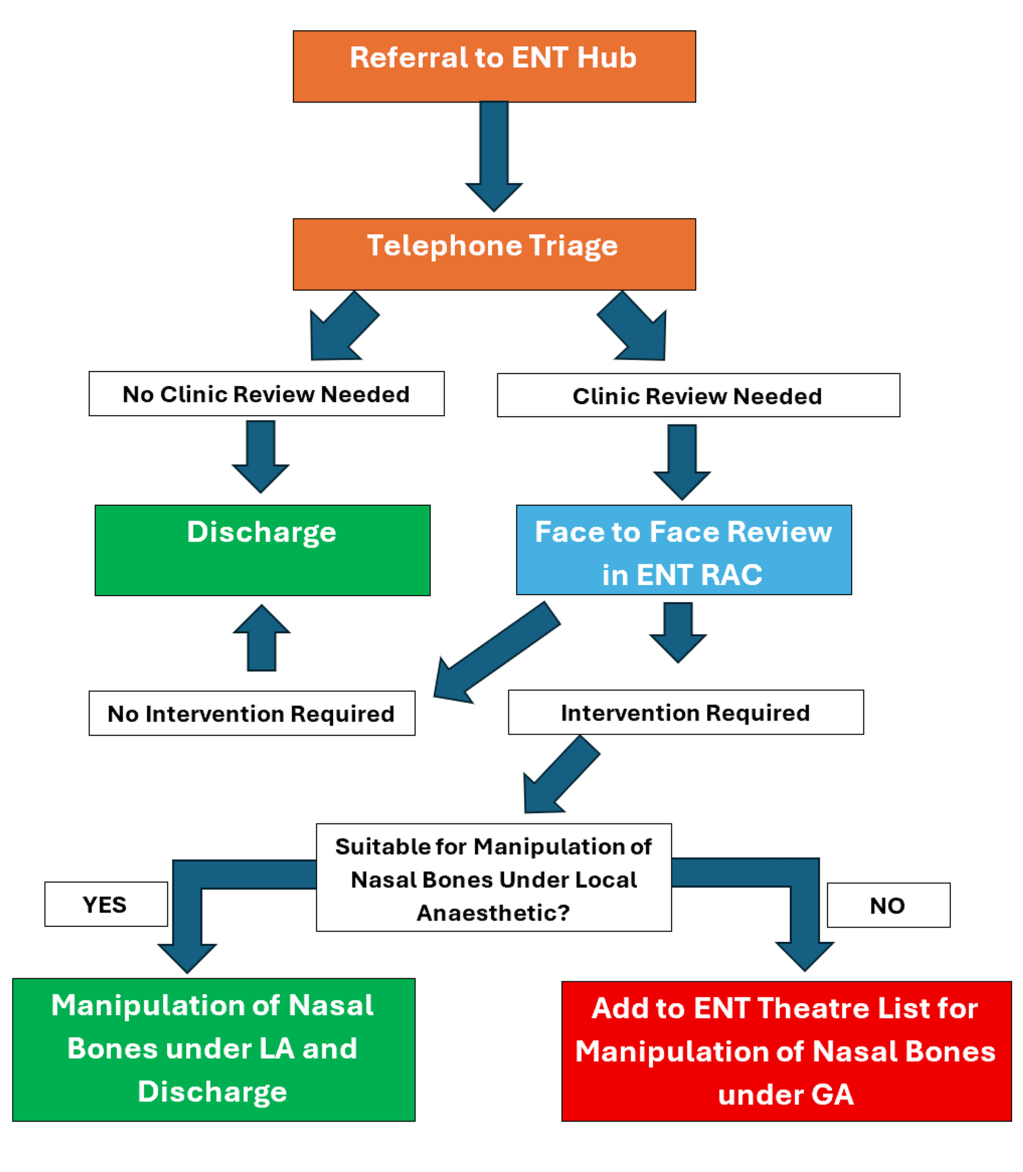
Patient Journey Flowchart

Patients were initially contacted via telephone by ENT clinicians approximately five to seven days after injury, to ensure initial swelling had reduced prior to triage. Based on history and symptoms obtained during this telephone consultation, patients were either reassured or invited for clinic assessment to evaluate their injury and the need for intervention. The threshold for warranting clinic review was intentionally low to ensure patients were not missed. Those with significant deformity, functional issues, or cosmetic concerns were reviewed face-to-face in a rapid access clinic and considered for MNB. If MNB was warranted and suitable to be carried out under local anaesthetic, this was performed at the same appointment. If a general anaesthetic was required, patients were added to the next available ENT theatre list (Figure 1).

## Results

A total of 56 referrals were analysed (age range: 7-89 years). The majority of injuries were due to falls and sporting activities, although mechanisms were not consistently documented.

Referral timelines

Fifty-two (93%) referrals were received within seven days of injury (range: 0-13 days). Initial patient contact was attempted for all patients, but only 35 (63%) were contacted within seven days of referral (Table 1).

Clinic review

Thirty-four (61%) patients were reviewed in clinic. Of these, 25 (74%) (Table 1) were seen within two weeks of injury (range: 6-21 days). Telephone triage avoided unnecessary clinic attendance for 22 (39%) patients referred.

Intervention

Nineteen (34%) patients referred required MNB. There was significant variation between age groups of those requiring MNB (Table 2). Of the 19 patients requiring MNB, 13 (68%) underwent the procedure within 14 days of injury, while six (32%) patients received delayed intervention (>14 days). Delays ranged from 6 to 26 days post-injury.

**Table 1:** Referral and Review Timelines Data are presented as mean (range) and n (%). Percentages are calculated from the total requiring the respective intervention (referral: 56; contact: 56; clinic review: 34; and manipulation of nasal bones (MNB): 19). Statistical significance was defined at p < 0.05.

Metric	Mean (Range)	Within Standard, N (%)
Injury to referral	2 days (0–13)	52 (93%)
Referral to contact	7 days (0–23)	35 (63%)
Injury to clinic review	13 days (6–24)	25 (74%)
Injury to MNB	13 days (6–26)	13 (68%)

**Table 2:** Outcomes by Age Group Data are presented as n (%). Percentages in the “Patients Referred” column are out of all 56 referrals. Percentages in the “Patients Requiring MNB” column are out of the referrals within that age group. Statistical analysis was performed with the chi-squared test and Fisher’s exact test; p < 0.05 was considered significant. The chi-squared test for association between age group and requirement for manipulation of nasal bones (MNB) showed χ²(4) = 3.47, p = 0.482, which was not statistically significant.

Age Group	Patients Referred, n (%)	Patients Requiring MNB, n (%)
<14 years	8 (14%)	3 (38%)
15–17	5 (9%)	2 (40%)
18–40 years	27 (48%)	11 (41%)
41–65 years	11 (20%)	3 (27%)
>65 years	5 (9%)	0 (0%)

Paediatric subgroup

**Figure 2:**
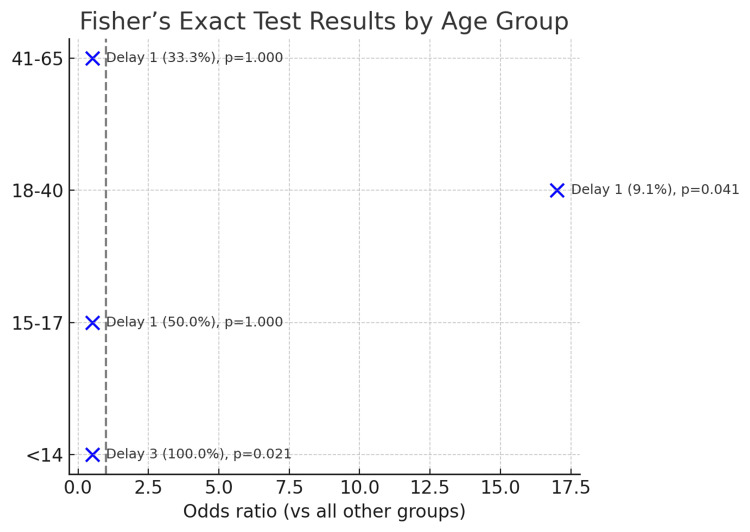
Age Group Analysis - Time to MNB Fisher’s exact test and delay to manipulation of nasal bones (MNB). Data are presented as odds ratios with 95% CI and delay rate N (%). Significance was defined as p < 0.05.

**Figure 3:**
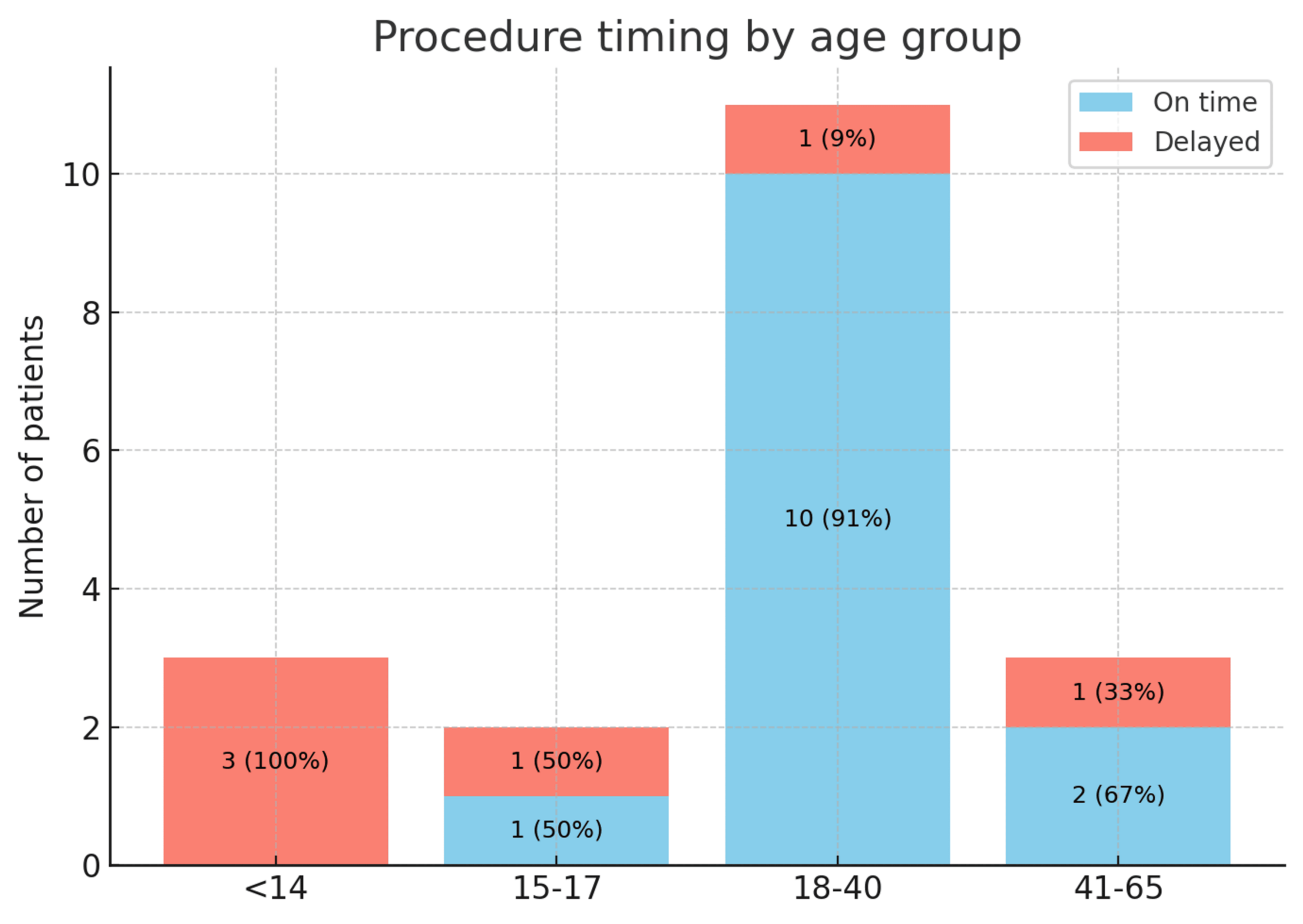
Procedure Timing by Age Group Stacked bar chart: procedures performed on time vs delayed by age group. Data are presented as absolute N. Delays are defined as >14 days post-injury. Significance is defined as p < 0.05.

All three (100%) paediatric patients (<14 years) requiring MNB under general anaesthetic exceeded the recommended 14-day timeframe due to limited theatre capacity. In contrast, 10 (91%) patients aged 18-40 years underwent intervention on time, reflecting that intervention in this age group tends to be more feasible under local anaesthetic and with greater patient engagement. These data are presented in Figures 2, 3. 

## Discussion

This audit highlights both strengths and areas for improvement in the current referral pathway for NBFs at Ulster Hospital, Dundonald. Referral receipt was generally prompt, with most referrals submitted within a week of injury. However, contact with patients occurred within a week for only 35 (63%) patients, indicating a potential bottleneck in service delivery.

Telephone triage reduced unnecessary clinic reviews by 22 (39%), consistent with reports that triage can be a safe and effective tool for identifying patients who require intervention [5,6]. A systematic review further supports that telemedicine optimises clinic volumes, though diagnostic accuracy may vary depending on the modality used [7]. Nonetheless, reliance on telephone-based assessment risks underestimating severity or missing injuries, particularly if patients are uncontactable or reluctant to answer unfamiliar numbers. Although there may be some knock-on delay if the initial telephone triage is delayed, this is minimised by the nature of the rapid access clinic, which normally has appointments available the following day, and by the prerogative of the clinician booking the clinics to overbook if necessary.

The paediatric subgroup revealed significant delays, with all three (100%) cases managed outside the recommended timeframe. Children are at greater risk of functional and cosmetic sequelae due to ongoing nasal development, and international guidelines recommend assessment and intervention within three to seven days after swelling resolution [4,8]. Early clinic assessment, ideally within five days, would facilitate more timely surgical planning for this cohort.

Age group analysis overall demonstrated variation in the proportion of patients requiring manipulation of nasal bones, ranging from 0/5 (0%) in those older than 65 years to 11/27 (41%) in the 18-40 year group. However, statistical testing demonstrated no significant association between age group and the likelihood of requiring MNB (χ²(4) = 3.47, p = 0.482). This suggests that while descriptive trends exist, age alone was not a determinant of intervention in this cohort. For future cycles, a trend analysis such as the Cochran-Armitage test could be considered to assess whether there is a linear relationship between increasing age and reduced intervention rates.

Interestingly, the 18-40 year group demonstrated both the highest requirement for manipulation (11/27 (41%)), which was not statistically significant, and relatively timely intervention compared with other age categories, which was statistically significant. This may reflect both patient-related and service-related factors, including greater health-seeking behaviour in this age range, fewer comorbidities delaying anaesthetic clearance, and a clinical perception that outcomes are particularly relevant for a socially and professionally active population. These findings suggest that younger adults are more likely to receive timely definitive management, whereas paediatric patients risk delay.

International audits confirm that delays in managing NBFs are common, with standards frequently unmet [9,10]. Our findings are consistent with these trends, emphasising the need for systemic improvements.

Future improvements should include dedicated paediatric clinic slots, clearer patient communication, and ensuring referral details contain accurate, accessible contact information. Consideration of video-based triage may also be valuable, with studies showing high patient satisfaction and timely identification of cases requiring manipulation. In some studies, the use of video or photographic consultations reduced the need for attendance by as much as 42% and also showed high sensitivity and specificity for those requiring intervention [11,12]. The vast majority of studies compare telephone triage and video triage to “face-to-face” appointments rather than to each other, although both have been shown to be effective separately. Remote consultations, however, should be balanced against evidence that they may increase follow-up requirements for new referrals compared with in-person review [13].

The limitations of this audit include its short duration (two and a half months) and relatively small sample size, which may not capture seasonal variations. Furthermore, incomplete documentation of injury mechanisms limited the scope of epidemiological analysis.

## Conclusions

Telephone triage is effective in reducing unnecessary clinic attendances for nasal bone fractures. However, delays in patient contact and paediatric surgical scheduling limited the timeliness of intervention. Prioritising early face-to-face assessment for children by day five post-injury, ensuring referral forms contain accurate and up-to-date contact details, and educating patients to expect calls from unrecognised numbers could all help minimise delays. In addition, allocating dedicated theatre capacity for paediatric nasal fractures would reduce avoidable postponements, while expanding future audits to cover a full year would provide a more comprehensive understanding of seasonal variation and service pressures.
